# Characterization of Histone H3 Lysine 4 and 36 Tri-methylation in *Brassica rapa* L.

**DOI:** 10.3389/fpls.2021.659634

**Published:** 2021-06-07

**Authors:** Hasan Mehraj, Satoshi Takahashi, Naomi Miyaji, Ayasha Akter, Yutaka Suzuki, Motoaki Seki, Elizabeth S. Dennis, Ryo Fujimoto

**Affiliations:** ^1^Graduate School of Agricultural Science, Kobe University, Kobe, Japan; ^2^RIKEN Center for Sustainable Resource Science, Yokohama, Japan; ^3^Department of Horticulture, Faculty of Agriculture, Bangladesh Agricultural University, Mymensingh, Bangladesh; ^4^Department of Computational Biology, Graduate School of Frontier Sciences, The University of Tokyo, Kashiwa, Japan; ^5^RIKEN Cluster for Pioneering Research, Saitama, Japan; ^6^Kihara Institute for Biological Research, Yokohama City University, Yokohama, Japan; ^7^CSIRO Agriculture and Food, Canberra, ACT, Australia; ^8^School of Life Sciences, Faculty of Science University of Technology, Sydney, NSW, Australia

**Keywords:** histone H3 lysine 4 tri-methylation, histone H3 lysine 36 tri-methylation, epigenetics, subfunctionalization, *brassica*

## Abstract

Covalent modifications of histone proteins act as epigenetic regulators of gene expression. We report the distribution of two active histone marks (H3K4me3 and H3K36me3) in 14-day leaves in two lines of *Brassica rapa* L. by chromatin immunoprecipitation sequencing. Both lines were enriched with H3K4me3 and H3K36me3 marks at the transcription start site, and the transcription level of a gene was associated with the level of H3K4me3 and H3K36me3. H3K4me3- and H3K36me3-marked genes showed low tissue-specific gene expression, and genes with both H3K4me3 and H3K36me3 had a high level of expression and were constitutively expressed. Bivalent active and repressive histone modifications such as H3K4me3 and H3K27me3 marks or antagonistic coexistence of H3K36me3 and H3K27me3 marks were observed in some genes. Expression may be susceptible to changes by abiotic and biotic stresses in genes having both H3K4me3 and H3K27me3 marks. We showed that the presence of H3K36me3 marks was associated with different gene expression levels or tissue specificity between paralogous paired genes, suggesting that H3K36me3 might be involved in subfunctionalization of the subgenomes.

## Introduction

*Brassica rapa* L. encompasses commercially important cultivars of vegetables, oilseeds, condiments, and fodder and is a crop closely related to *Arabidopsis thaliana* (Cheng et al., 2014, 2016a; Lv et al., 2020). In addition to its agronomic significance, *B. rapa* is also important for genomic studies, because it has the first complete genome sequence to be determined within the genus *Brassica* (Wang et al., 2011). *B. rapa* (AA genome) is one of the ancestral species of the allotetraploid species, *Brassica nigra* L (AABB) and *Brassica napus* L (AACC) (Chalhoub et al., 2014; Yang et al., 2016). The *B. rapa* genome has undergone a whole-genome triplication after speciation between *A. thaliana* and *B. rapa* (Wang et al., 2011). The whole-genome triplication results in multiple copies of paralogous genes and generates three subgenomes, the least fractioned subgenome (LF) and two more fractionated subgenomes (MF1 and MF2) within the *B. rapa* genome (Wang et al., 2011). After the whole genome triplication, subfunctionalization such as different expression levels or DNA methylation levels among three subgenomes or paralogous genes has been observed (Parkin et al., 2014; Chen et al., 2015).

The basic unit of chromatin is the nucleosome, which consists of 147 bp of DNA wrapped around a histone octamer containing two of each of H2A, H2B, H3, and H4 (Li et al., 2007). Chromatin structure is regulated by posttranslational modification of histone proteins such as methylation, acetylation, phosphorylation, ubiquitylation, and sumoylation (Strahl and Allis, 2000; Jenuwein and Allis, 2001; Li et al., 2007). Specific amino acid residues of the N-terminal tail of histone proteins are targets for posttranslational modifications that can impact gene expression by altering the chromatin structure. In plants, tri-methylation of histone H3 lysine 4 (H3K4me3) and H3K36me3 are often associated with transcriptional activation and that of H3K9me2 and H3K27me3 with transcriptional repression (Fuchs et al., 2006; Li et al., 2007; Xiao et al., 2016). Histone modification is an epigenetic regulatory mechanism, which affects transcriptional activity of chromatin without changing DNA sequence and is crucial for the development and the adaptation of plants to changing environments (Fujimoto et al., 2012; Quadrana and Colot, 2016).

The application of high-throughput sequencing technologies provides an opportunity to identify the genome-wide profiles of histone modification by a combination of chromatin immunoprecipitation (ChIP) and genome-wide sequencing (ChIP-seq). With ChIP-seq, the genome-wide distribution patterns of histone modifications such as H3K4me3, H3K9me2, H3K27me3, and H3K36me3 have been identified in some plant species. In *A. thaliana*, H3K4me3, H3K27me3, and H3K36me3 were found in euchromatin, while H3K9me2 was found in heterochromatin (Turck et al., 2007; Zhang et al., 2007, 2009; Bernatavichute et al., 2008; Roudier et al., 2011). In *A. thaliana*, H3K4me3 and H3K36me3 marked highly expressed genes, while H3K27me3 marked lowly expressed genes or genes showing tissue-specific expression (Turck et al., 2007; Zhang et al., 2007, 2009; Roudier et al., 2011). In *B. rapa*, H3K9me2 was associated with transcriptional repression of genes and was overrepresented in transposable elements (TEs) (Takahashi et al., 2018). H3K27me3 also showed an association with transcriptional repression of genes and a role in tissue- or developmental stage-specific transcriptional regulation (Akter et al., 2019; Payá-Milans et al., 2019; Shen et al., 2019).

Despite whole-genome epigenome information in *B. rapa* having been obtained for repressive histone marks such as H3K9me2 or H3K27me3 (Takahashi et al., 2018; Akter et al., 2019; Payá-Milans et al., 2019), there is no report of the whole epigenome information for active histone marks. In the present study, we examined the distribution of H3K4me3 and H3K36me3 using two inbred lines of Chinese cabbage, RJKB-T23 and RJKB-T24, and investigated the role of H3K4me3 and H3K36me3 in transcription, tissue-specific gene expression, subfunctionalization of paralogous genes, and species conservation and response to biotic and abiotic challenges.

## Materials and Methods

### Plant Materials and Growth Conditions

Two Chinese cabbage inbred lines (*B. rapa* var. *pekinensis*), RJKB-T23 (T23) and RJKB-T24 (T24), were used (Kawamura et al., 2016). The genetic distances between the two lines (T23 and T24) and the reference genome were similar, suggesting that the genetic distance between the two lines was moderate among Chinese cabbage accessions (Kawamura et al., 2016; Shea et al., 2018). Seeds were surface-sterilized and grown on agar-solidified Murashige and Skoog (MS) plates with 1% (w/v) sucrose under long-day (LD) conditions (16 h light) at 22°C. First and second leaves were harvested at 14 days after sowing for ChIP analyses, and the plant phenotype at this stage in the two lines was shown in Akter et al. (2019).

### ChIP-seq

ChIP experiments were performed as described by Buzas et al. (2011). One gram of first and second leaves of *B. rapa* was used for ChIP analysis, and anti-H3K4me3 (Millipore, 07-473) and H3K36me3 (Abcam, ab9050) antibodies were used. Before ChIP-seq, we validated the enrichment of purified immunoprecipitated DNAs by qPCR using the positive and negative control primer sets of H3K4me3 and H3K36me3 previously developed (Supplementary Figure S1) (Kawanabe et al., 2016). Purified immunoprecipitated DNA and input DNA were sequenced by HiSeq 2000 (36 bp single end). These sequence data have been submitted to the DDBJ database^1^ under accession number DRA003120. Low-quality reads or adapter sequences were purged from the ChIP-seq reads using Cutadapt version 1.7.1 and Trim Galore! version 0.3.7. The reads were mapped to the *B. rapa* reference genome v.1.5^2^ using Bowtie 2 version 2.2.3 (Supplementary Table S1). The mapped reads on the interspersed repeat regions (IRRs), such as the TEs detected by RepeatMasker, were examined (Supplementary Table S1). For sequential ChIP, experiments were performed as described by Finnegan et al. (2011). Anti-H3K4me3 antibodies were used for first ChIP, and anti-H3K27me3 antibodies (Millipore, 07-449) were used for second ChIP.

ChIP-qPCR was performed using a LightCycler Nano (Roche). The immunoprecipitated DNA was amplified using FastStart Essential DNA Green Master (Roche). PCR conditions were 95°C for 10 min followed by 40 cycles of 95°C for 10 s, 60°C for 10 s, and 72°C for 15 s, and melting program (60°C to 95°C at 0.1°C/s). After amplification cycles, each reaction was subjected to melt temperature analysis to confirm single amplified products. Data presented are the average and standard error (s.e.) from three biological and experimental replications. Enrichment of H3K4me3 or H3K36me3 marks was calculated by comparing the target gene and non-H3K4me3- or non-H3K36me3-marked genes, respectively, by qPCR using immunoprecipitated DNA as a template. Bra011336 that did not have H3K4me3 and H3K27me3 marks was used for reference for sequential ChIP-qPCR for examining enrichment of target genes. The difference between primer pairs was corrected by calculating the difference observed by qPCR amplifying the input DNA as a template. Primer sequences used in this study are shown in Supplementary Table S2.

### Detection of H3K4me3 and H3K36me3 Peaks by Model-Based Analysis for ChIP-seq

We performed peak calling on alignment results using MACS 2 2.1.0 and identified the regions having H3K4me3 or H3K36me3 peaks. The MACS callpeak was used with the options (effective genome size: 2.30e + 08, band width: 200, model fold: 10–30, tag size: 36) described by Akter et al. (2019). A *p*-value cutoff of 1.00e−05 was used to consider peaks significant. H3K4me3- and H3K36me3-marked genes were defined as genes that had a more than 200-bp-length peak within a genic region (exon-intron) including 200 bp upstream and downstream as described by Akter et al. (2019).

### Gene Ontology Analysis

Analysis for enrichment of gene functional ontology terms was completed using the gene ontology (GO) tool agriGO (Du et al., 2010) following the methods described by Shimizu et al. (2014). Statistical tests for enrichment of functional terms used the hypergeometric test and false discovery rate (FDR) correction for multiple testing to a level of 1% FDR.

## Results

### Identification of H3K4me3- and H3K36me3-Marked Genes in *B. rapa*

**Figure 1:**
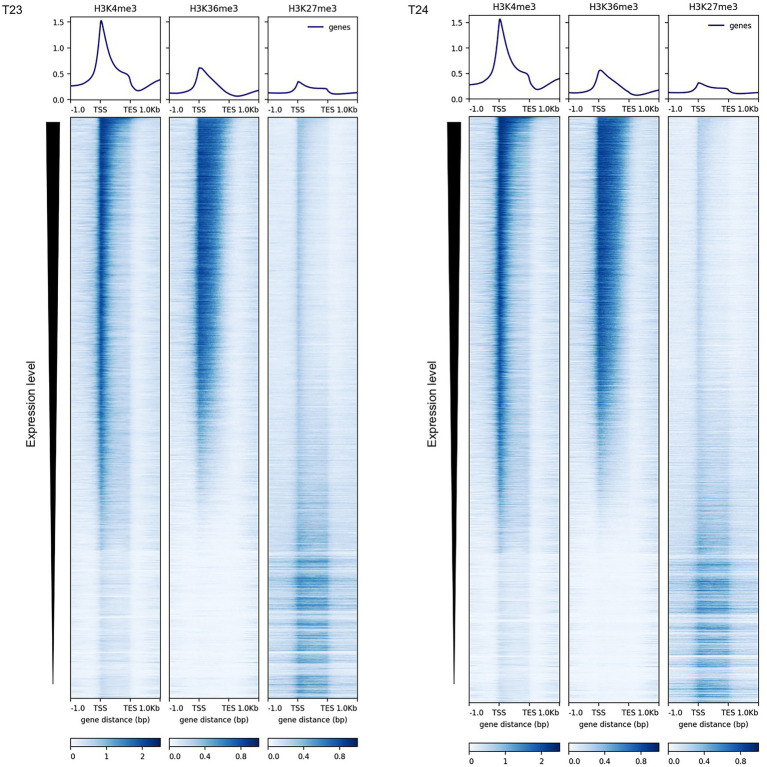
Distribution of H3K4me3, H3K36me3, and H3K27me3 marks in the genic regions in RJKB-T23 (T23) and RJKB-T24 (T24). The deepTools version 3.1.3 is used for visualization (https://deeptools.readthedocs.io/en/develop/).

The impact of the active histone marks, H3K4me3 and H3K36me3, on gene expression in *B. rapa*; their interaction with other epigenetic marks; and the diversity and conservation of H3K4me3 and H3K36me3 distribution between *B. rapa* and *A. thaliana* were examined. The presence of H3K4me3 or H3K36me3 marks on the chromatin of 14-day leaves in two inbred lines of Chinese cabbage (RJKB-T23 (T23) and RJKB-T24 (T24)) was mapped by ChIP-seq (Supplementary Table S1). Reads mapped in the genic regions were classified into 2 kb upstream, exon, intron, and 2 kb downstream segments. The proportion of reads in exons was slightly higher than in the input DNA (Supplementary Figure S2 and Supplementary Table S3), suggesting that there is preferential location of H3K4me3 and H3K36me3 in exon regions. H3K4me3 and H3K36me3 were enriched in the transcribed regions in both lines, especially around the transcription start sites (TSS) (Figure 1 and Supplementary Figure S3). The percentage of H3K4me3 and H3K36me3 reads in the IRRs (TEs and repeats) was lower than in the input DNA in both lines (Supplementary Table S1). There was no H3K4me3 and H3K36me3 enrichment in IRR sequences or their flanking regions (Supplementary Figure S3).

We defined an H3K4me3- or H3K36me3-marked gene as having a peak of more than 200 bp within the genic region, which includes the 200 bp upstream and downstream sequences (see section “Materials and Methods”). In T23 and T24, 18,475 (46.6%) and 19,208 genes (48.5%) had H3K4me3 marks, respectively, and 16,759 genes were common to the two lines and were termed H3K4me3-marked genes (Supplementary Tables S4 and S5). In T23 and T24, 13,395 (33.8%) and 13,771 genes (34.8%) had H3K36me3 marks, respectively, and 11,844 genes were common to the two lines and were termed H3K36me3-marked genes (Supplementary Tables S4 and S5).

Previously obtained SNP data for T23 and T24 were used (Shea et al., 2018; Akter et al., 2019). Genes having H3K4me3 or H3K36me3 marks tended to have a higher SNP number per length in each gene (mutation rate) than genes without H3K4me3 or H3K36me3 marks, respectively, in T23 and T24 (Supplementary Figure S4). However, there was no difference between genes having H3K4me3 or H3K36me3 marks and the total genes (Supplementary Figure S4).

### Identification of Genes Carrying Bivalent or Antagonistic Active and Repressive Histone Modification

**Figure 2:**
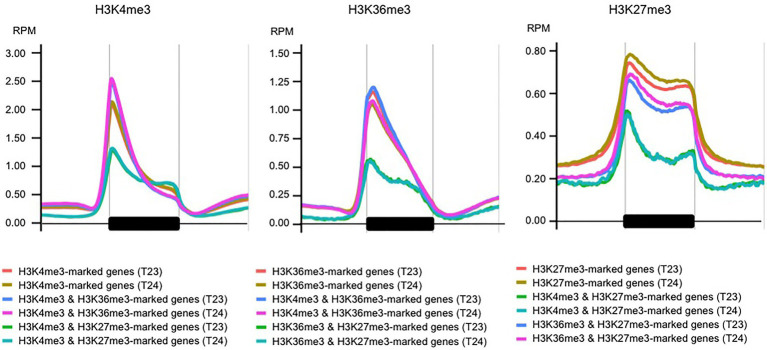
Metagene plots of H3K4me3, H3K36me3, and H3K27me3 in genic regions. H3K4me3 **(left panel)**, H3K36me3 **(middle panel)**, and H3K27me3 **(right panel)** levels at the genic region with 1 kb upstream and 1 kb downstream are shown using genes having one or two histone modifications.

We counted the overlapped genes among three histone marks (H3K4me3 and H3K36me3 in this study and H3K27me3 in Akter et al., 2019). Of the H3K36me3-marked genes, 85.4% (*n* = 10,119) also had H3K4me3 marks (Supplementary Tables S4–S6). The enrichment of H3K4me3 or H3K36me3 marks was similar between genes having one or two modifications (Figure 2). These results suggest that these two modifications were in the same region of each gene. Three functional *BrFLC* paralogs (*BrFLC1*, *BrFLC2*, and *BrFLC3*) had H3K4me3 and H3K36me3 marks (Supplementary Figure S5).

Of the H3K27me3-marked genes, 6.4% (*n* = 671) had H3K36me3 (Supplementary Tables S4 and S5); there was antagonistic coexistence of active and repressive histone modifications between H3K36me3 and H3K27me3 marks. Two of the three *BrSOC1* (Bra004928 and Bra039324) were induced by 4 weeks of cold treatment (Shea et al., 2019). All three *BrSOC1* paralogs had the H3K27me3 mark but not the H3K4me3 or H3K36me3 mark (Supplementary Figure S5). Previously, we identified genes showing a difference in H3K27me3 levels between 2-day cotyledons and 14-day leaves in both T23 and T24 (Akter et al., 2019). In 903 genes showing a higher H3K27me3 level in 2-day cotyledons than in 14-day leaves, 549 genes (60.8%) had H3K36me3 marks in 14-day leaves (Supplementary Table S5). In 395 genes showing a higher H3K27me3 level in 14-day leaves than in 2-day cotyledons, only 10 genes (2.5%) had H3K36me3 marks in 14-day leaves (Supplementary Table S5). In 21 genes categorized into “post-embryonic development” with higher H3K27me3 levels in 14-day leaves than in 2-day cotyledons, including *BrFUS3*, *BrDOC1*, and *LEA* genes, none of the 21 genes had H3K36me3 marks in 14-day leaves, suggesting that increasing H3K27me3 may be antagonistic to H3K36me3 accumulation.

**Figure 3:**
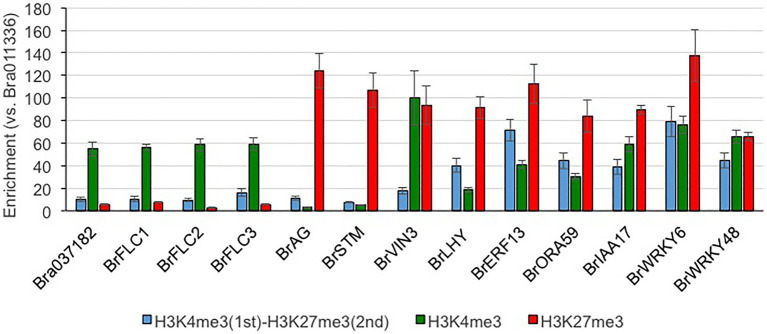
Sequential ChIP-qPCR in RJKB-T24. Bra011336 that does not have H3K4me3 and H3K27me3 is used as reference gene for qPCR. Bra037182 and three *BrFLC* paralogs are H3K4me3-marked genes, and *BrAG* and *BrSTM* are H3K27me3-marked genes. *BrVIN3*, *BrLHY*, *BrERF13*, *BrORA59*, *BrIAA17*, *BrWRKY6*, and *BrWRKY48* had both H3K4me3 and H3K27me3 marks by ChIP-seq, and distribution of these modification in each gene was shown in Supplementary Figures S5, S9. Values are means ± standard error (s.e.; three biological and technical replicates) of relative H3K4me3, H3K27me3, or H3K4me3/H3K27me3 levels.

Of the H3K27me3-marked genes, 35.4% (*n* = 3,699) had H3K4me3; there were bivalent active and repressive histone modifications between H3K4me3 and H3K27me3 marks (Supplementary Tables S4 and S5). There are two *BrVIN3* paralogs. *BrVIN3a* (Bra020445) was induced by 4 weeks of cold treatment, while *BrVIN3b* (Bra006824) was not (Shea et al., 2018). *BrVIN3a* had both H3K4me3 and H3K27me3 marks, while *BrVIN3b* had neither H3K4me3 nor H3K27me3 marks (Supplementary Figure S5). In genes having both H3K4me3 and H3K27me3 marks, accumulation of the H3K4me3 mark was flat in the genic region, and the H3K4me3 level around the TSS was lower than that in H3K4me3-marked genes (Figure 2 and Supplementary Figure S6). The H3K27me3 level in genes having both H3K4me3 and H3K27me3 marks was lower than that in H3K27me3-marked genes, especially in the middle-to-3' part of the genic region (Figure 2 and Supplementary Figure S6). The level of H3K36me3 mark in the genes having both H3K36me3 and H3K27me3 marks was also lower than that in H3K36me3-marked genes, while accumulation of the H3K27me3 mark in the genes having both H3K36me3 and H3K27me3 marks was similar to that in H3K27me3-marked genes (Figure 2 and Supplementary Figure S7). More than 30 and 80% of genes having both H3K4me3 and H3K27me3 marks had overlapped peaks with more than 500 and 150 bp in length, respectively (Supplementary Table S6), suggesting that different histone modifications tended to detect the same position on the gene. In case of genes having both H3K36me3 and H3K27me3 marks, more than 15 and 55% of genes had overlapped peaks with more than 500 and 150 bp in length (Supplementary Table S6). In genes having both H3K4me3 and H3K27me3 marks, the categories related to transcriptional regulation were highly overrepresented including transcription factors such as *LFY*, *WRKY*, *ERF*, *IAA*, and *HFSB* (Supplementary Figures S8, S9 and Supplementary Table S7). To examine the simultaneous occupancy of active (H3K4me3) and repressive (H3K27me3) histone modifications, sequential ChIP-qPCR in 14-day leaves in T24 was performed in seven genes having both H3K4me3 and H3K27me3 marks detected by ChIP-seq analysis. Six of the seven genes showed enrichment for the second modification compared with genes having only the H3K4me3 or H3K27me3 mark (Figure 3). *BrVIN3* showed lower enrichment than the other six genes having both H3K4me3 and H3K27me3 marks (Figure 3).

### H3K36me3 Marks Were Associated With a Transcriptionally Active State and Constitutive Expression

**Figure 4:**
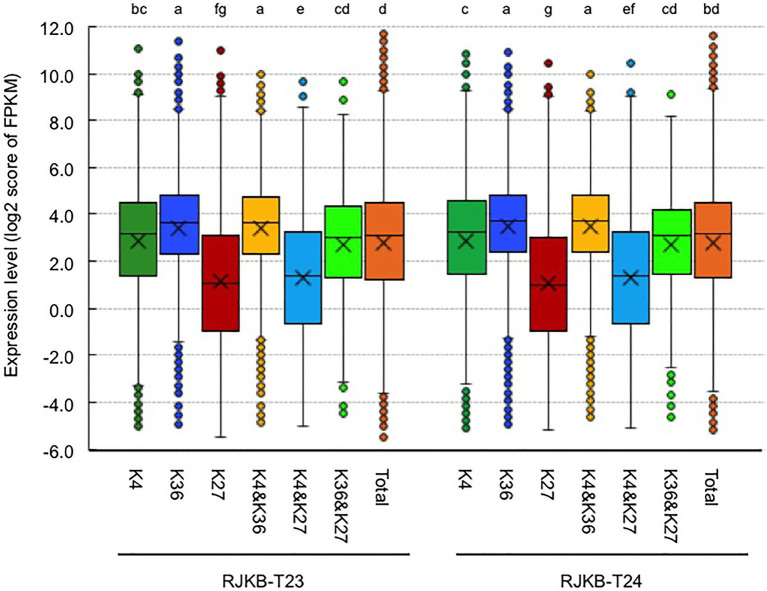
Box plots of the gene expression levels of the log 2 score of FPKM with H3K4me3 (K4), H3K36me3 (K36), and H3K27me3 (K27) in genic regions of RJKB-T23 and RJKB-T24. “&” represents genes having two different histone marks. “Total” indicates the log 2 score of FPKM in all genes (FPKM < 0.01). FPKM: fragments per kilobase of transcript per million mapped reads. Different letters indicate significant difference (Tukey’s HSD test, *p* < 0.05).

The average transcription level of H3K4me3- or H3K36me3-marked genes was higher than that in the total genes, and the average transcription level of H3K4me3-marked genes was lower than that in H3K36me3-marked genes (Figure 4). The average transcription level of genes having both H3K4me3 and H3K36me3 marks showed higher expression levels than the total genes (Figure 4). The average transcription level of genes having both H3K4me3 and H3K27me3 marks or having both H3K36me3 and H3K27me3 marks showed a lower expression level than H3K4me3- or H3K36me3-marked genes, respectively, and the decreased average expression level of genes also having H3K27me3 was larger in H3K4me3-marked genes than in H3K36me3-marked genes; genes with bivalent active and repressive histone modifications, H3K4me3 and H3K27me3 marks, showed similar expression levels to those with the repressive histone modification, H3K27me3 (Figure 4).

**Figure 5:**
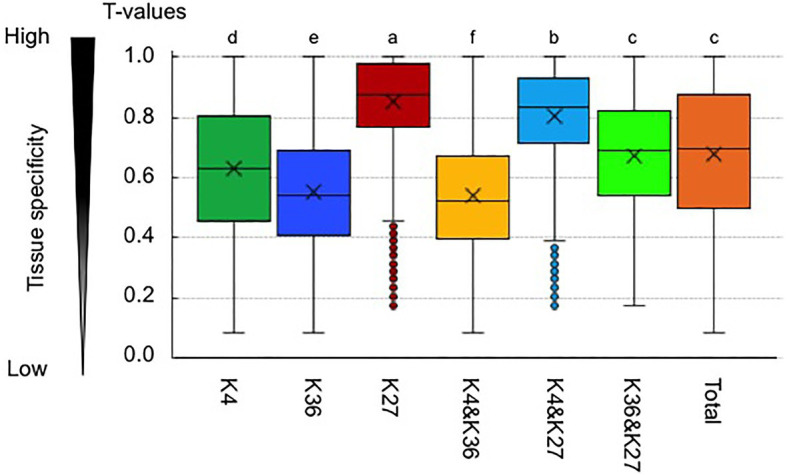
Tissue specificity of expression in genes having an H3K4me3 (K4) or H3K36me3 (K36) mark. A tissue specificity index, *T*-value, which interpolates the entire range between 0 for housekeeping genes and 1 for strictly one-tissue-specific genes, was calculated using the transcriptome data in six different tissues in *B. rapa*. K27 represents the H3K27me3-marked genes. “&” represents genes having two different histone marks. “Total” indicates *T*-value in all genes. Different letters indicate significant difference (Tukey’s HSD test, *p* < 0.05).

Previously, we calculated a tissue specificity index, *T*-value, which interpolates the entire range between 0 for housekeeping genes and 1 for strictly one-tissue-specific genes, using the transcriptome data from six different tissues in *B. rapa* (Tong et al., 2013; Akter et al., 2019). We found that H3K36me3-marked genes and genes having both H3K4me3 and H3K36me3 marks showed significantly lower average *T*-values compared with the total genes (Figure 5), suggesting that H3K36me3 has a role in constitutive gene expression. H3K4me3-marked genes had lower average *T*-values compared with total genes, but the *T*-value was not as low as in H3K36me3-marked genes. H3K4me3 or H3K36me3 genes also marked with H3K27me3 showed increased average *T*-values, and this effect was greater in H3K4me3-marked genes than in H3K36me3-marked genes (Figure 5); bivalent active and repressive histone modifications, H3K4me3 and H3K27me3 marks, showed a higher tissue specificity similar to the H3K27me3-marked genes.

### Bivalent Active and Repressive Histone Modifications, H3K4me3 and H3K27me3, Increased Transcriptional Sensitivity in Stress Response

**Figure 6:**
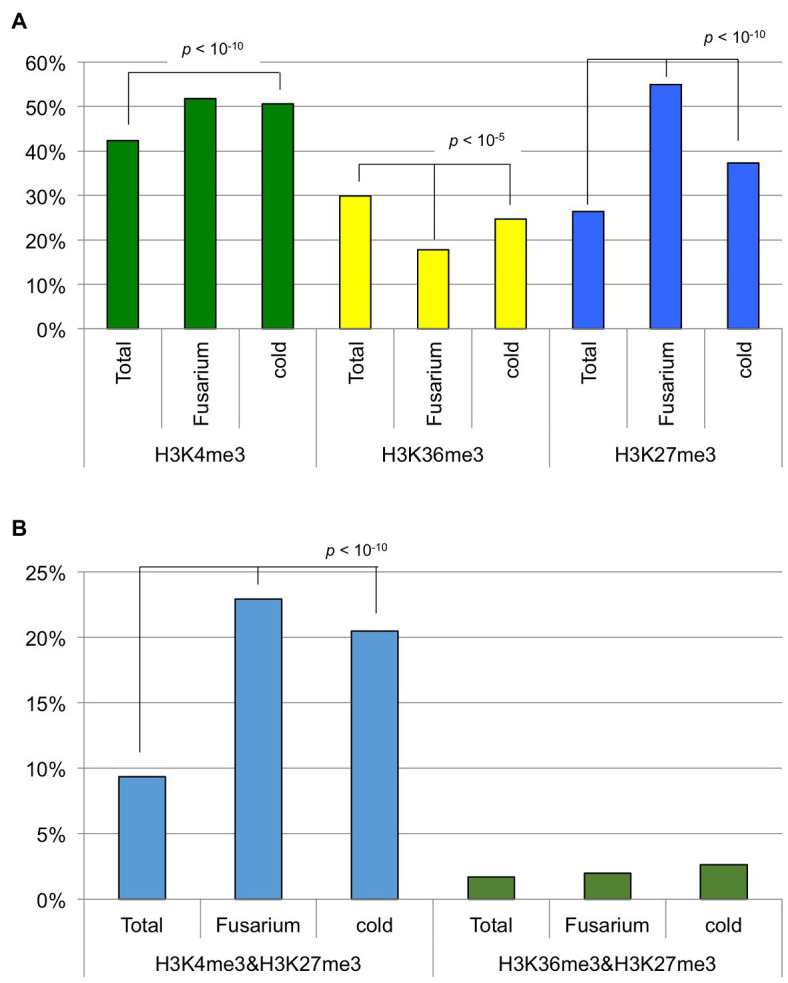
The percentage of genes having histone modifications. **(A)** Genes having H3K4me3, H3K36me3, or H3K27me3 marks were used. **(B)** Genes having both H3K4me3 and H3K27me3 marks or both H3K36me3 and H3K27me3 marks were used. “Total” represents all the annotated genes in *B. rapa*. “Fusarium” and “cold” represent genes differentially expressed following *Fusarium oxysporum* f. sp. *conglutinans* inoculation and 4 weeks of cold treatment (vernalization), respectively.

In this study, 42.3, 29.9, and 26.4% of annotated genes were defined as H3K4me3-, H3K36me3-, and H3K27me3-marked genes. Previously, we identified genes in T24 that were differentially expressed following *Fusarium oxysporum* f. sp. *conglutinans* (*Foc*) inoculation or 4 weeks of cold treatment (vernalization) compared with non-treated samples (Miyaji et al., 2017; Shea et al., 2019). We examined whether the genes with H3K4me3, H3K27me3, and H3K36me3 marks showed changed expression caused by these two stress treatments. Of 253 differentially expressed genes following *Foc* inoculation, 131 (51.8%), 45 (17.8%), and 139 (54.9%) genes had H3K4me3, H3K36me3, and H3K27me3 marks, respectively (Figure 6). Of 1,441 differentially expressed genes resulting from 4 weeks of cold treatment, 729 (50.6%), 356 (24.7%), and 538 (37.7%) genes had H3K4me3, H3K36me3, and H3K27me3 marks, respectively (Figure 6). These results showed that genes with altered expression in both stress treatments had a significantly lower percentage of H3K36me3-marked (chi-squared test, *p* < 10^−5^) and a higher percentage of H3K27me3-marked genes (chi-squared test, *p* < 10^−10^) than in total genes, suggesting that H3K36me3-marked genes tended to be transcriptionally stable and H3K27me3-marked genes tended to be variable in response to these two stress treatments.

Of the annotated genes, 9.3% had both H3K4me3 and H3K27me3 marks, and 1.7% had both H3K36me3 and H3K27me3 marks. Of 253 genes differentially expressed following *Foc* inoculation, 58 (22.9%) genes had both H3K4me3 and H3K27me3 marks, and five (2.0%) genes had both H3K36me3 and H3K27me3 marks (Figure 6). Of 1,441 genes differentially expressed following 4 weeks of cold treatment, 295 (20.5%) genes had both H3K4me3 and H3K27me3 marks, and 38 (2.6%) genes had both H3K36me3 and H3K27me3 marks (Figure 6). These results suggest that genes having both H3K4me3 and H3K27me3 marks tended to have changed levels of transcription associated with the two stress treatments, but genes having both H3K36me3 and H3K27me3 marks did not have changed transcription levels.

### Comparison of H3K4me3 and H3K36me3 States Between Paralogous Genes in *B. rapa*

We compared H3K4me3 or H3K36me3 locations between paralogs. Among the 1,675 three-copy sets, 392 had H3K4me3 in all three copies, 317 had H3K4me3 in at least two copies, 364 had H3K4me3 in at least one copy, and 602 sets did not have H3K4me3 in any copies (Supplementary Figure S10). In the case of H3K36me3, 235 had H3K36me3 in all three copies, 242 had H3K36me3 in at least two copies, 333 had H3K36me3 in at least one copy, and 865 sets did not have H3K27me3 in any copies (Supplementary Figure S10).

**Figure 7:**
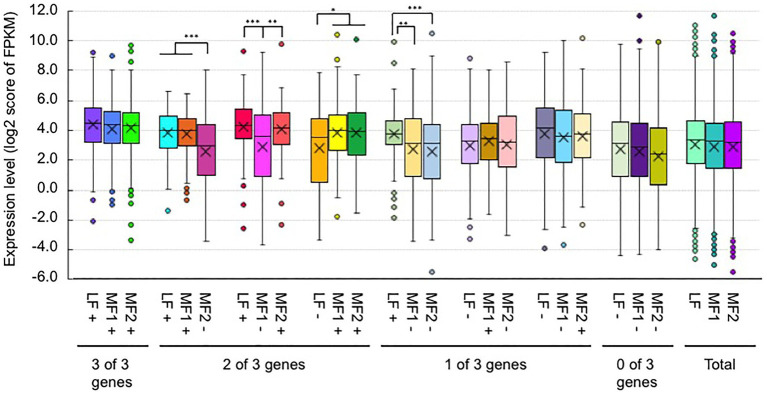
Comparison of the expression level (FPKM) between paralogous pairs with and without H3K36me3 marks in RJKB-T23. Values are means ± standard error (s.e.) of FPKM. “+” and “−” represent the presence and absence of H3K36me3 marks, respectively. ^*^p < 0.05; ^**^p < 0.01; ^***^p < 0.001 (Student *t*-test).

We examined whether a difference in H3K4me3 or H3K36me3 states between paralogs was associated with a different level of gene activity. Between paralogous pairs, there was no significant difference of the average expression levels between genes with and without H3K4me3 marks in either T23 or T24 (Supplementary Figures S11, S12). Between paralogous pairs, the average expression levels of genes with H3K36me3 marks tended to be higher than those without H3K36me3 in both lines, and some comparisons showed significantly different expression levels between paralogous pairs with and without H3K36me3 marks (Figure 7 and Supplementary Figure S13), indicating that the presence of H3K36me3 is associated with a difference of gene expression level between paralogous pairs.

**Figure 8:**
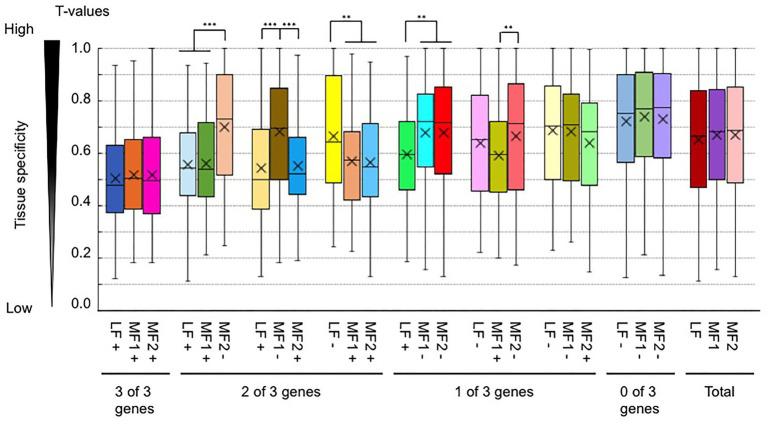
Comparison of the tissue specificity of expression (a tissue specificity index, *T*-value) between paralogous pairs with and without H3K36me3 marks. Values are means ± standard error (s.e.) of FPKM. “+” and “−” represent the presence and absence of H3K4me3 marks, respectively. ^**^p < 0.01; ^***^p < 0.001 (Student *t*-test).

We also examined whether a difference in H3K4me3 or H3K36me3 states between paralogs was associated with a different level of *T*-value. *T*-values between paralogs with and without H3K4me3 marks tended to be the same (Supplementary Figure S14), while the average *T*-values of genes with H3K36me3 tended to be lower than those without H3K36me3 marks when paralogous pairs were compared (Figure 8), suggesting an association of H3K36me3 with constitutive gene expression.

### Relationship Among Epigenetic Marks

We compared H3K4me3 or H3K36me3 distribution to the other epigenetic marks, H3K9me2, H3K27me3, and DNA methylation (Takahashi et al., 2018; Akter et al., 2019). At the whole-genome level, there was high positive correlation between H3K4me3 and H3K36me3, while there was a negative correlation between H3K4me3 or H3K36me3 and DNA methylation (Supplementary Figure S15). In genic regions or IRRs, there was a positive correlation between H3K4me3 and H3K36me3, but the relationship between H3K4me3 or H3K36me3 and DNA methylation was not clear. In IRRs, there was a positive relationship among four histone modifications (*r* = 0.40–0.79), but there was no relationship between the presence of the four histone modifications and DNA methylation (*r* = −0.04–0.15; Supplementary Figure S15).

The distribution of H3K4me3 and H3K36me3 was similar, with a partial overlap with H3K27me3, but it is antagonistic to the distribution of H3K9me2 and DNA methylation (Supplementary Figures S16, S17).

The average level of DNA methylation in the region overlapping H3K4me3 or H3K36me3 regions was lower than in the total genome (Supplementary Figure S18), indicating that H3K4me3 or H3K36me3 regions were preferentially not DNA methylated.

### Half of the Genes Marked With H3K4me3 or H3K36me3 Are Shared Between *B. rapa* and *A. thaliana*

To gain information about conservation of H3K4me3 and H3K36me3 states beyond *B. rapa*, we compared the genes marked with H3K4me3 and H3K36me3 in *B. rapa* and *A. thaliana*. About 50% of H3K4me3- or H3K36me3-marked genes in *A. thaliana* overlapped with at least one orthologous gene in *B. rapa* (Supplementary Table S8). This percentage is higher than that in H3K27me3-marked genes (Supplementary Table S8). These overlapped genes obtained using a data set by Engelhorn et al. (2017) were termed species-conserved H3K4me3- or H3K36me3-marked genes.

## Discussion

We examined the genomic locations of H3K4me3 and H3K36me3 using two *B. rapa* lines. Since the results in the two lines were quite similar, this study focused on the genes having H3K4me3 or H3K36me3 in both lines as biological replicates, which could suggest a more fundamental epigenetic state in *B. rapa*. About 47 and 34% of total genes had H3K4me3 and H3K36me3 marks, respectively, and these percentages are similar to the case in *A. thaliana* (H3K4me3, 50–70%; H3K36me3, −50%) (Luo et al., 2013; Sequeira-Mendes et al., 2014; Engelhorn et al., 2017), suggesting that our analysis does not overestimate the number of genes having these histone marks. Both H3K4me3 and H3K36me3 marks were enriched close to the TSS, with a sharper peak in H3K4me3 than in H3K36me3. These distribution patterns of H3K4me3 and H3K36me3 marks in the genic region are similar to those in *A. thaliana* and rice (Roudier et al., 2011; Liu et al., 2019). Low levels of H3K4me3 and H3K36me3 marks were observed in IRRs, especially in the body region.

H3K4me3 and H3K36me3 are known as active histone marks, and H3K4me3- and H3K36me3-marked genes showed higher expression levels on average than the total gene expression levels. With RNA-seq data from six tissues, a tissue-specific index, *T*-value, has been developed, and *T*-values indicate a range between 0 for housekeeping or constitutive genes and 1 for genes showing tissue-specific expression (Tong et al., 2013). The average *T*-value of H3K4me3- or H3K36me3-marked genes was lower than that of total genes, suggesting that H3K4me3- and H3K36me3-marked genes had a low level of tissue specificity, being constitutively expressed. Of the two active histone modifications, the H3K36me3 mark was more associated with higher expression levels and lower tissue specificity. A similar trend was observed in response to *Foc* inoculation and 4 weeks of cold treatment; H3K36me3-marked genes are more transcriptionally stable than H3K4me3-marked genes in response to these two stress treatments.

We compared H3K4me3 and H3K36me3 to other epigenetic modifications. The H3K4me3 and H3K36me3 marks showed a negative correlation with DNA methylation and H3K9me2 at the whole genome level. DNA methylation levels in the regions having H3K4me3 or H3K36me3 were low. These results indicate that H3K4me3 and H3K36me3 do not physically coexist with the transcriptional repression mark of heterochromatin. A correlation between H3K4me3 or H3K36me3 and DNA methylation or H3K9me2 has also been identified in other plant species (Zhang et al., 2009; He et al., 2010; Roudier et al., 2011). Only 1.7% of the total genes (671 genes) had both H3K36me3 and H3K27me3 marks, suggesting that H3K36me3 marks were not compatible with H3K27me3 marks. Antagonistic roles for H3K36me3 and H3K27me3 were observed in the transcriptional regulation of *FLC* during vernalization (Yang et al., 2014), and in this study, antagonistic modification of H3K4me3/H3K36me3 and H3K27me3 in pre-vernalized plants was observed in three *BrFLC* paralogs. A higher level of H3K4me3 marks and lower level of H3K27me3 marks at *ABA INSENSITIVE 3* (*ABI3*), *LEAFY COTYLEDON 1/2* (*LEC1/2*), and *FUSCA3* (*FUS3*) levels increased their expression to regulate somatic embryo development in the *set domain group 8* (*sdg8*) and *embryonic flower 2* (*emf2*) double mutant in *A. thaliana* seedlings (Tang et al., 2012). In *A. thaliana*, changes of the active histone H3K4me3 mark and the repressive histone H3K27me3 mark have been observed in *DELAY OFGERMINATION1* (*DOG1*) during seed dormancy cycling; in dormant seeds, the H3K4me3 mark accumulated in *DOG1*, while the H3K4me3 mark was decreased and the H3K27me3 mark was increased during loss of dormancy and the germination process (Footitt et al., 2015; Cheng et al., 2020). In 21 genes that showed higher H3K27me3 levels in 14-day leaves than in 2-day cotyledons and categorized into “post-embryonic development” including *BrDOG1* and *BrFUS3* (Akter et al., 2019), no genes had H3K36me3 marks, suggesting that antagonistic H3K36me3 and H3K27me3 modifications might be important for the regulation of embryogenic expression of these genes.

The distribution of H3K4me3 or H3K36me3 partially overlapped with H3K27me3, and we identified genes with bivalent histone modification. The number of genes having both H3K4me3 and H3K36me3 marks was twice the expected number, suggesting that H3K4me3 and H3K36me3 preferentially co-localized. This is consistent with previous reports in *A. thaliana* (Luo et al., 2013; Sequeira-Mendes et al., 2014; Xi et al., 2020). Coexistence of the H3K4me3 and H3K36me3 marks at *SUPPRESSOR OF OVEREXPRESSION OF CO 1* (*SOC1*) chromatin in *A. thaliana* activates its expression (Berr et al., 2015), suggesting that coexistence of active marks may have an important role in transcriptional regulation during plant development. The numbers of genes having H3K4me3 and H3K27me3 marks or H3K36me3 and H3K27me3 marks were less than 20% or 80% of the expected number, respectively. However, about 9% of the total genes (3,699 genes) had both H3K4me3 and H3K27me3 marks in *B. rapa*. In these genes, a lower accumulation of H3K4me3 and H3K27me3 marks than in genes having either single modification was observed. The genes having H3K36me3 and H3K27me3 marks showed similar characteristics to the H3K36me3-marked genes such as having a high level of expression and a constitutive expression pattern. In contrast, the genes having H3K4me3 and H3K27me3 marks showed similar characteristics to H3K27me3-marked genes such as the low level of gene expression, highly tissue-specific gene expression, and overrepresentation in the category of “regulation of transcription.” Coexistence of H3K4me3 and H3K27me3 in the same chromosome fiber has been found in *A. thaliana*, and this was confirmed by sequential ChIP analysis (Sequeira-Mendes et al., 2014). In this study, we showed that some transcription factor genes have a simultaneous presence of H3K4me3 and H3K27me3 marks using sequential ChIP-qPCR. In *A. thaliana*, *VIN3* has a simultaneous presence of H3K4me3 and H3K27me3 marks (Finnegan et al., 2011). ChIP-seq analysis showed that *BrVIN3a* has both H3K4me3 and H3K27me3 marks, but sequential ChIP-qPCR did not show the simultaneous presence of these modifications. There were differences between *BrVIN3a* and *BrVIN3b* in the transcriptional response following vernalization and the histone modification in pre-vernalized states. By examining changes in these histone modifications in response to vernalization, the differing role of *VIN3* between species or between paralogs will be clarified. It has been suggested that the presence of both H3K4me3 and H3K27me3 marks may be alternative states of transcription in different tissues and/or stages in cell differentiation in *A. thaliana* (Sequeira-Mendes et al., 2014). In this study, genes having both H3K4me3 and H3K27me3 marks showed high tissue-specific gene expression and high sensitivity of transcription in response to biotic (*Foc* inoculation) and abiotic (4 weeks of cold treatment) stresses, suggesting that bivalent active and repressive histone modifications, H3K4me3 and H3K27me3, play a role in rapid response of transcription, not only through development but also following stress exposure.

*B. rapa* has experienced whole-genome triplication and has three subgenomes (LF, MF1, and MF2). There is a biased gene expression, distribution of TEs, and DNA methylation level among the three subgenomes in *B. rapa* (Cheng et al., 2012, 2016b; Chen et al., 2015). Genes covering LF tended to have a higher expression level than their paralogous genes covering MF1 or MF2, and DNA methylation level in LF was lower than that in MF1 or MF2 (Chen et al., 2015). The difference of expression levels between paralogous paired genes was associated with differences of H3K36me3 levels but not of H3K4me3. Previously, we have shown an association between gene expression levels and H3K27me3 levels between paralogous paired genes (Akter et al., 2019). The variation of the tissue specificity (*T*-values) between paralogous paired genes was associated with a difference of H3K36me3 or H3K27me3 levels (Akter et al., 2019). It is difficult to determine whether the relationship between histone modifications and transcription level is a cause or a consequence, but these results suggest that both active and repressive histone modifications, H3K36me3 and H3K27me3, play a role in the variation of gene expression between paralogous paired genes, which may be involved in subfunctionalization. Further analysis will be needed to verify this possibility.

## Data Availability Statement

The sequence data have been submitted to the DDBJ database (http://www.ddbj.nig.ac.jp) under accession numbers DRA003120 and DRA007199.

## Author Contributions

RF, HM, NM, and AA carried out the experiments. ST carried out the bioinformatics and statistical analysis of ChIP-seq data. RF, YS, MS, and ED planned the experiments. HM, ED, and RF wrote the manuscript. All authors contributed to the article and approved the submitted version.

### Conflict of Interest

The authors declare that the research was conducted in the absence of any commercial or financial relationships that could be construed as a potential conflict of interest.

## Footnotes

^1^http://www.ddbj.nig.ac.jp

^2^http://brassicadb.cn/
