# Supplementary material for: Characterization of Histone H3 Lysine 4 and 36 Tri-methylation in Brassica rapa L
Source: Front Plant Sci. 2021 Jun 7;12:659634. doi: 10.3389/fpls.2021.659634 (PMC8215614; doi:10.3389/fpls.2021.659634)
Supplement: Supplementary file 1 [file Data_Sheet_1.PDF]

Supplementary Material

Supplementary Figures

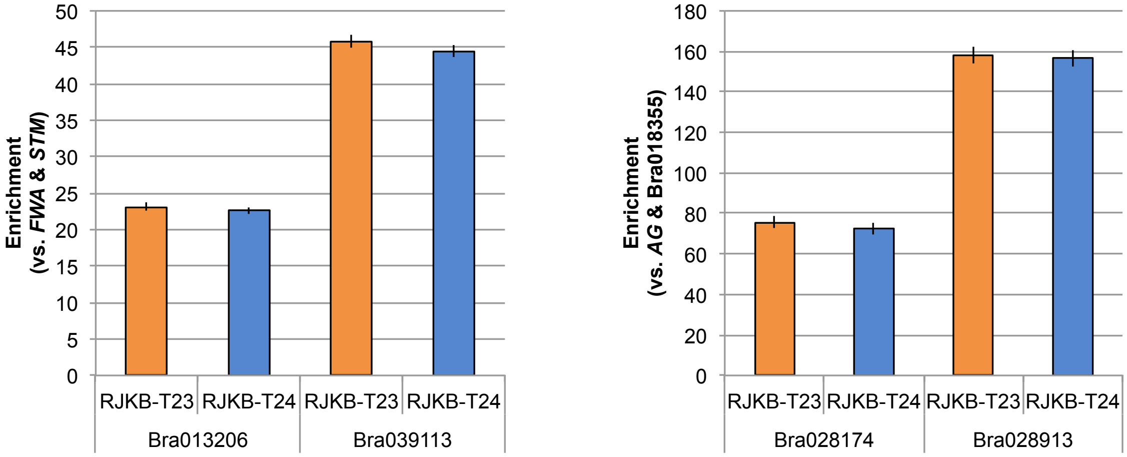

Supplementary Figure S1. Validation of the enrichment of purified immunoprecipitated (IP) DNAs by qPCR. Bra013206 and Bra039113 are H3K4me3-marked genes (positive control), and *FWA* and *STM* are low level H3K4me3-marked genes (negative control) (Kawanabe et al., 2016). Bra028174 and Bra028913 are H3K36me3-marked genes (positive control), and *AG* and Bra018355 are low level H3K36me3-marked genes (negative control) (Kawanabe et al., 2016). Values are means ± standard error (s.e.) (three biological and technical replicates) of relative H3K4me3 or H3K36me3 levels.

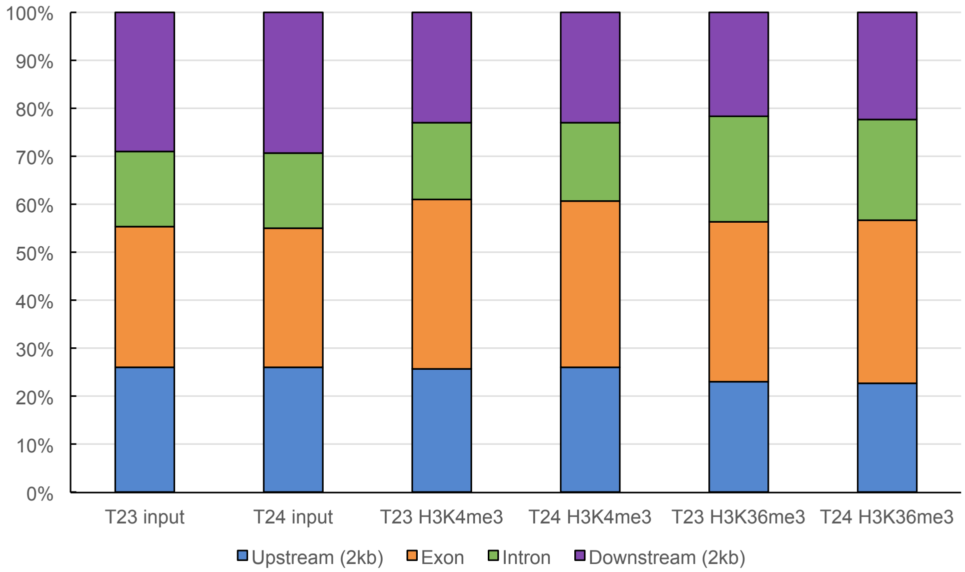

Supplementary Figure S2. Mapped reads of input and immunoprecipitated DNA using anti-H3K4me3 or anti-H3K36me3 antibodies in genic regions classified into 2kb-upstream, exon, intron, and 2kb downstream positions.

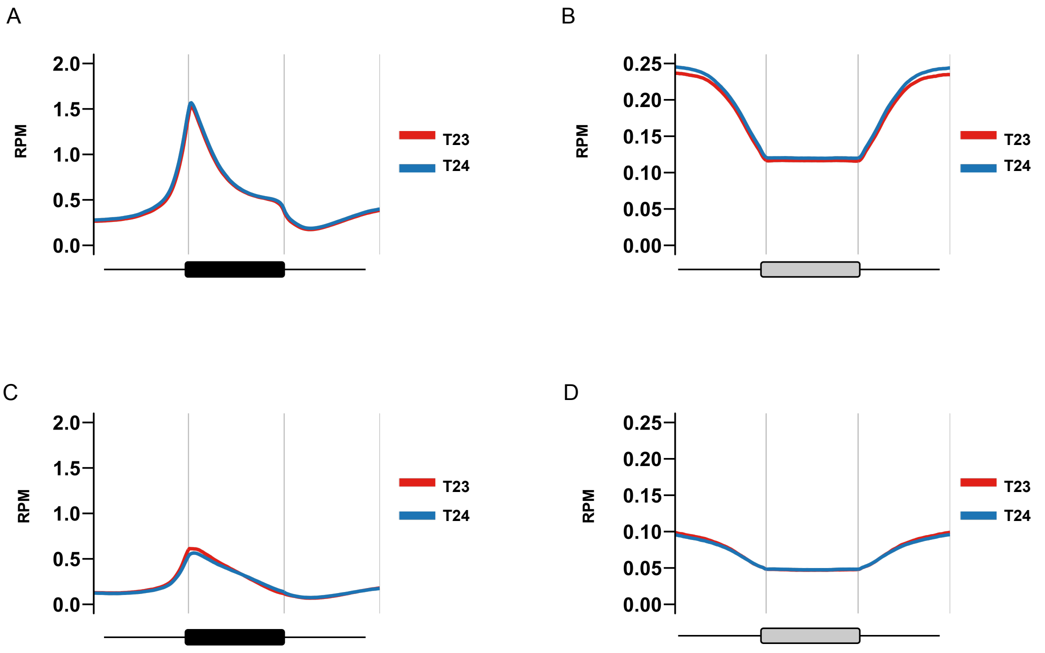

Supplementary Figure S3. Mapped reads of immunoprecipitated DNA using anti-H3K4me3 (A, C) or anti-H3K36me3 (B, D) antibodies in genic regions (black boxes) and IRR regions (gray boxes).

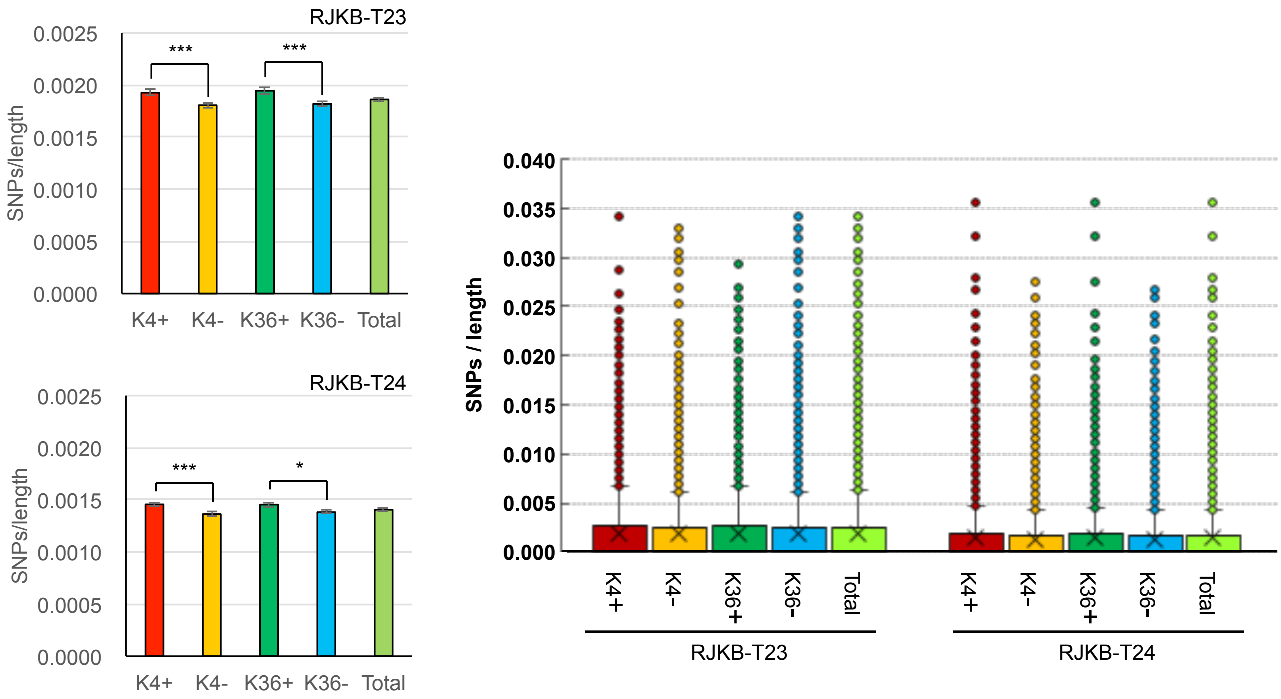

Supplementary Figure S4. Comparison of the average mutation rate (SNPs per length) in each gene having an H3K4me3 or H3K36me3 mark in RJKB-T23 and RJKB-T24. Values are means ± standard error (s.e.) of SNP numbers per length. *, $p < 0.05$, ***, $p < 0.001$ (Student *t*-test). '+' and '-' represent the presence and absence of H3K4me3/H3K36me3 marks in genes, respectively.

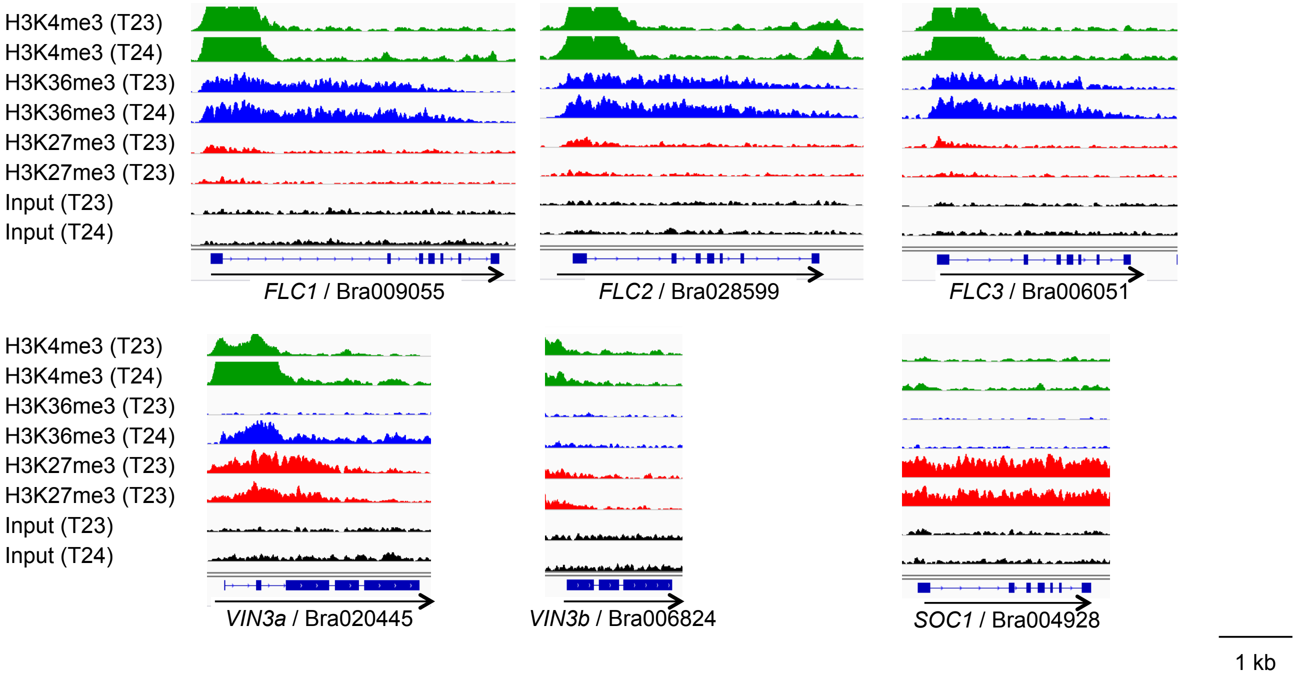

Supplementary Figure S5. Visualization of H3K4me3, H3K36me3, and H3K27me3 peaks by Integrative Genomics Viewer (IGV) by ChIP-seq.

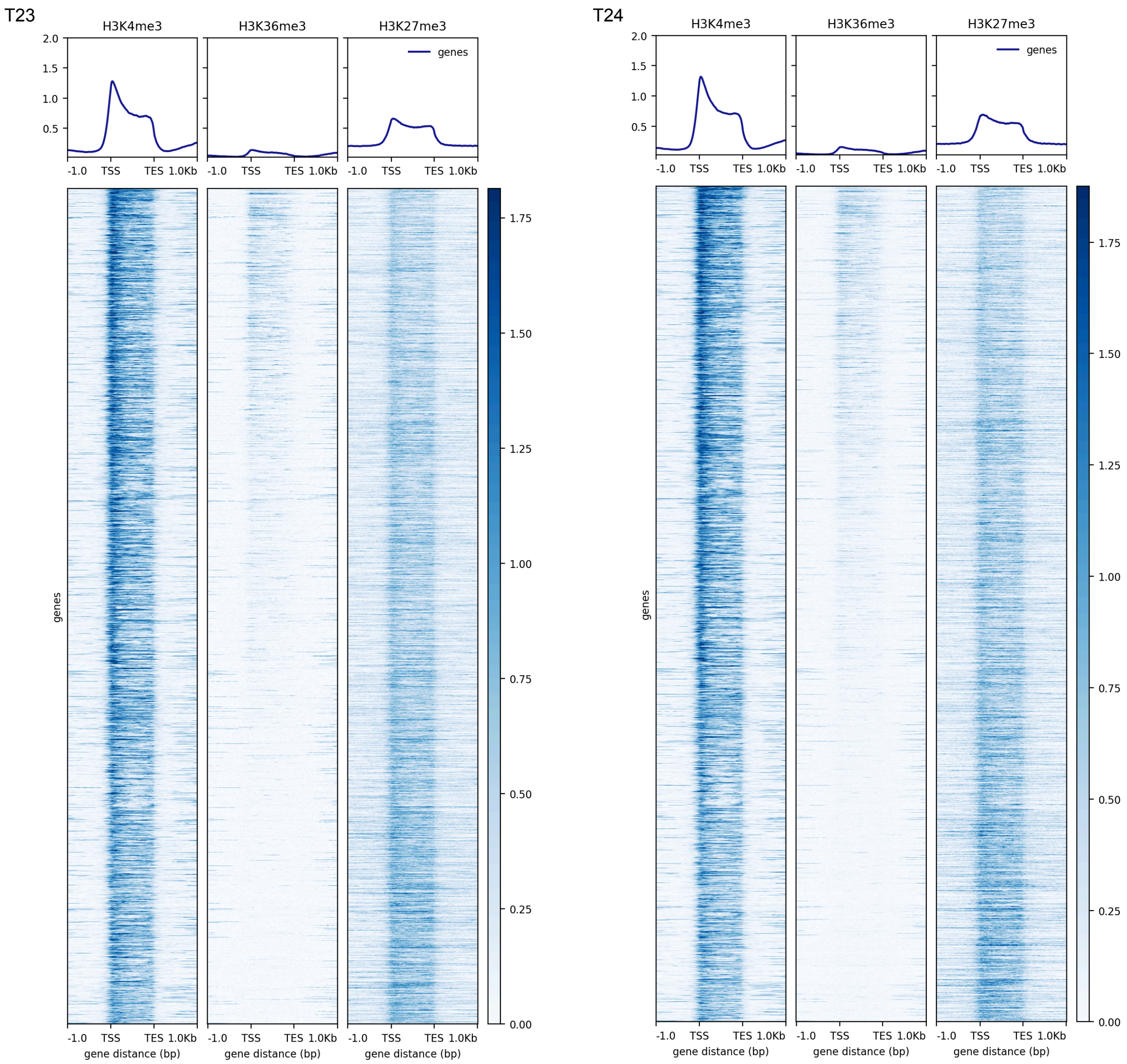

Supplementary Figure S6. Distribution of H3K4me3, H3K36me3, and H3K27me3 marks in the genic regions in RJKB-T23 (T23) and RJKB-T24 (T24). The genes having both H3K4me3 and H3K27me3 marks were used. The deeptools version 3.1.3 is used for visualization (https://deeptools.readthedocs.io/en/develop/).

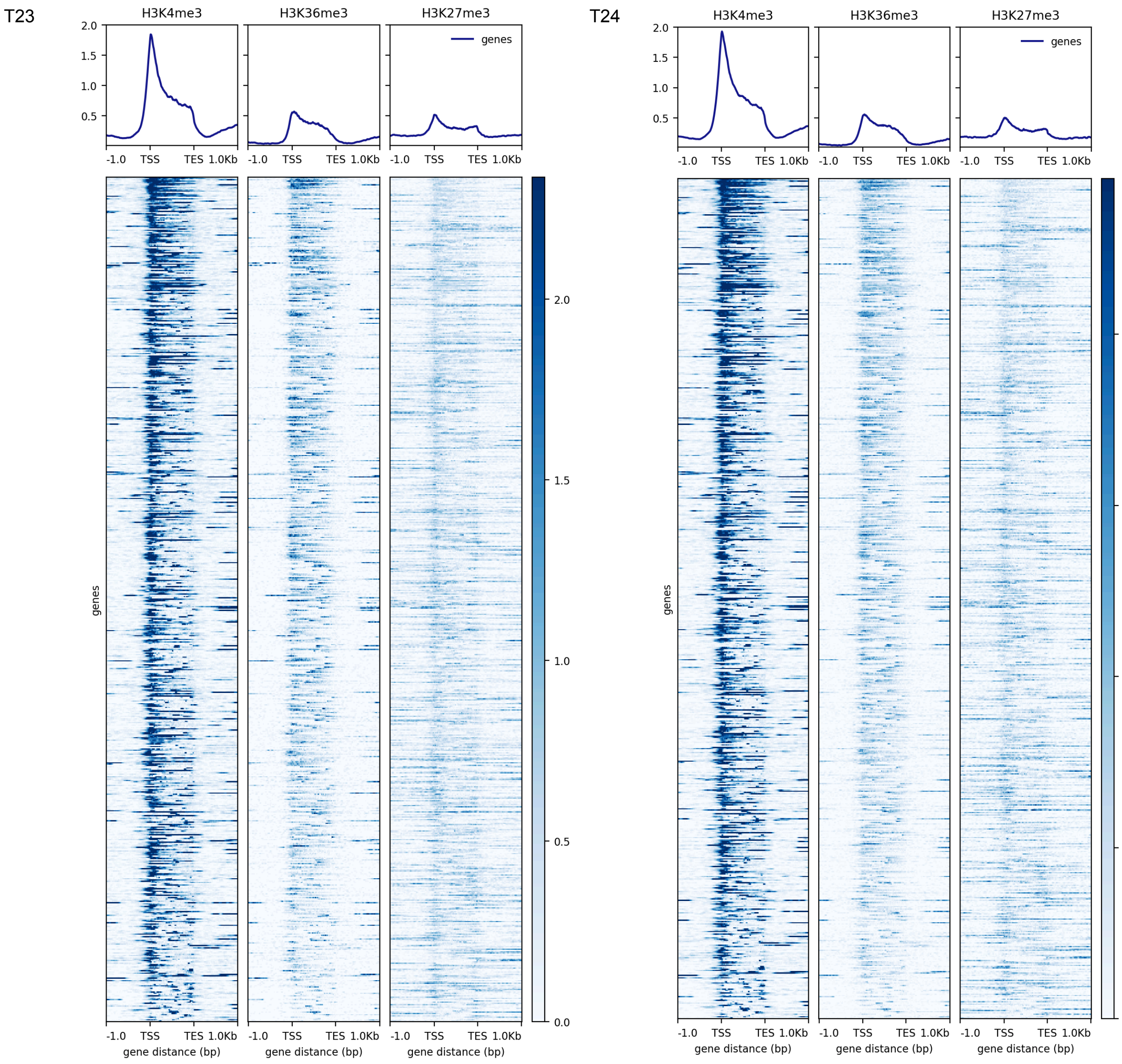

Supplementary Figure S7. Distribution of H3K4me3, H3K36me3, and H3K27me3 marks in the genic regions in RJKB-T23 (T23) and RJKB-T24 (T24). The genes having both H3K36me3 and H3K27me3 marks were used. The deeptools version 3.1.3 is used for visualization (https://deeptools.readthedocs.io/en/develop/).

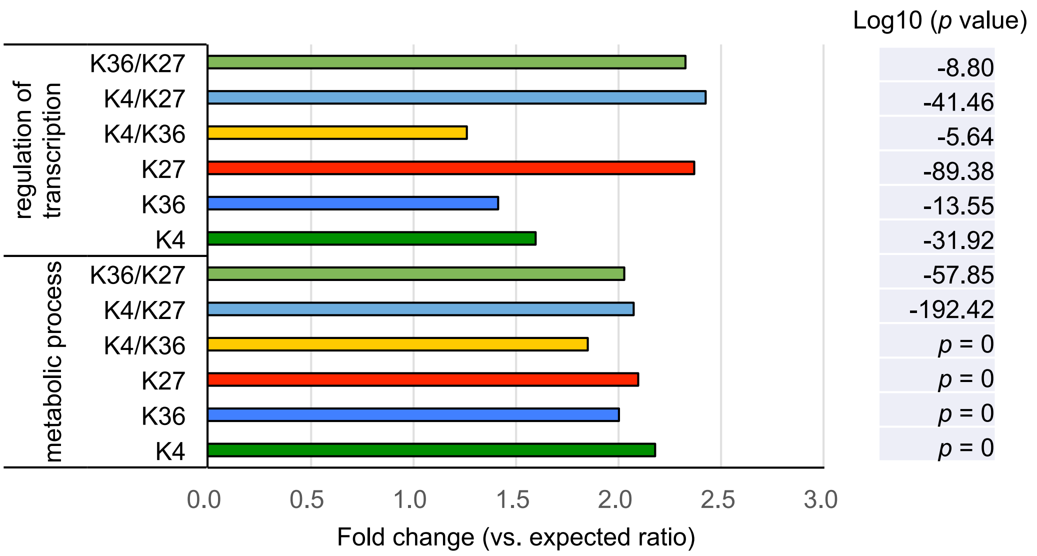

Supplementary Figure S8. GO classification in the category of 'regulation of transcription' and 'metabolic process'. The ratio was calculated by the percentage of genes having histone marks dividing the percentage of all annotated genes.

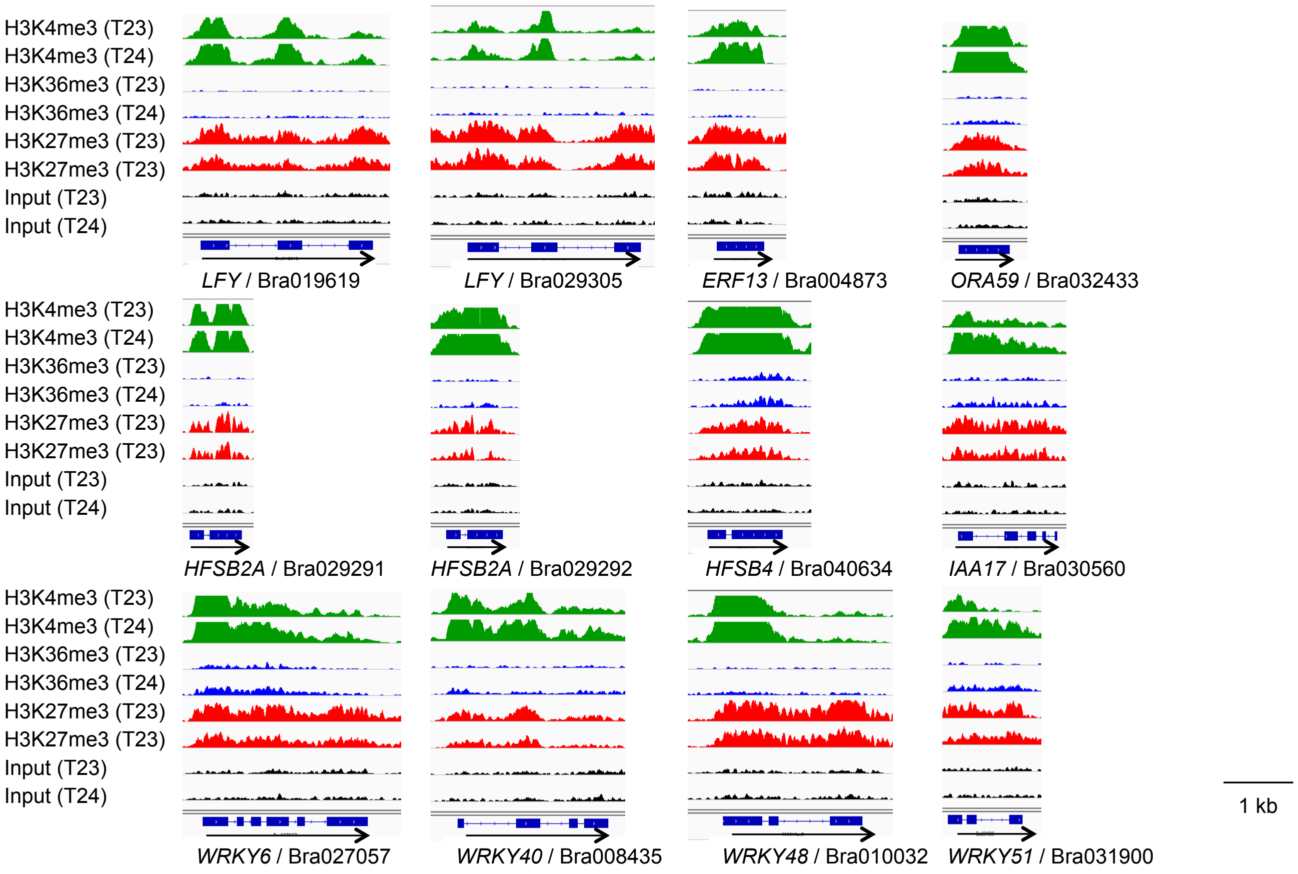

Supplementary Figure S9. Visualization of H3K4me3, H3K36me3, and H3K27me3 peaks in genes having H3K4me3 and H3K27me3 marks by Integrative Genomics Viewer (IGV) by ChIP-seq.

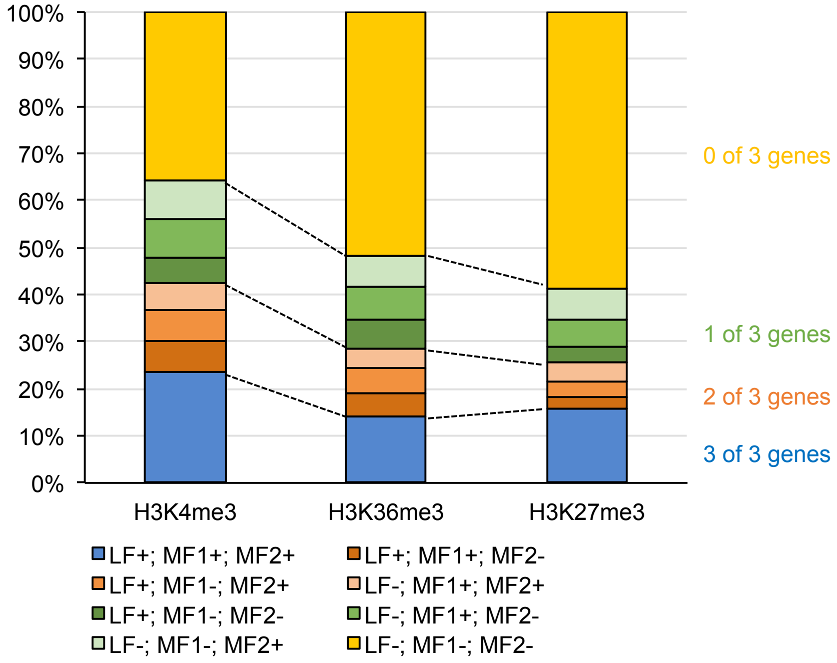

Supplementary Figure S10. Number of genes having H3K4me3 and H3K36me3 marks among three paralogous genes. '+' and '-' represent presence and absence of histone marks, respectively.

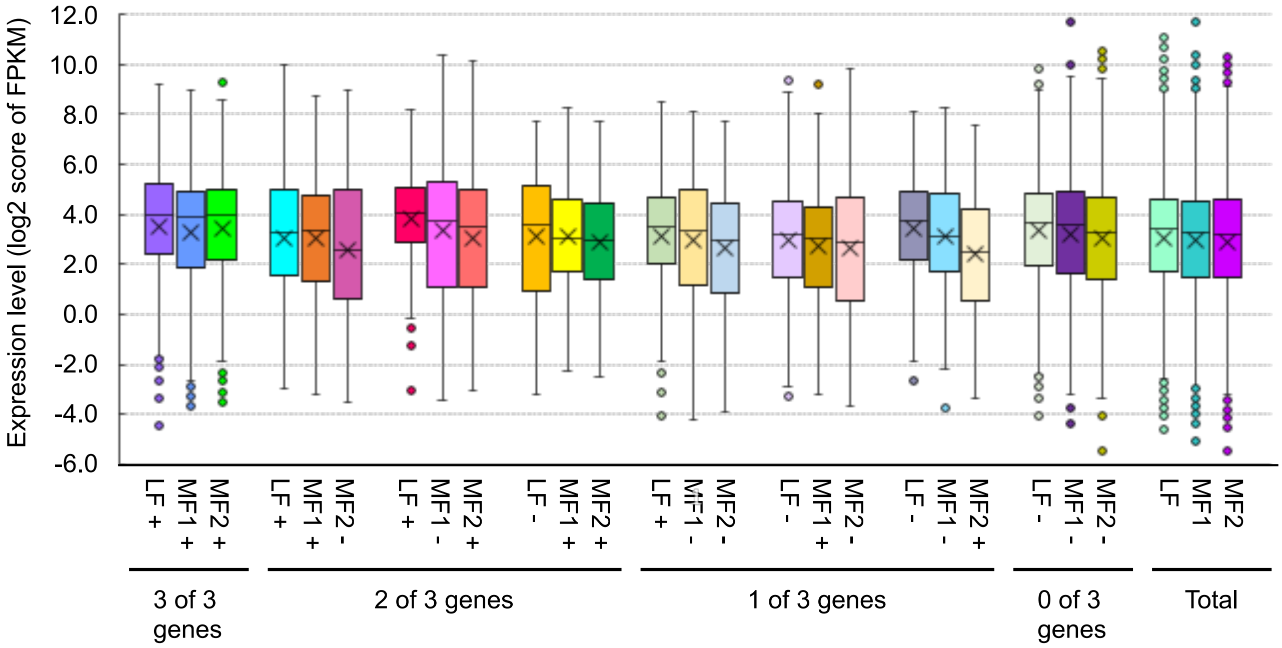

Supplementary Figure S11. Comparison of the expression level (FPKM) between paralogous pairs with and without H3K4me3 marks in RJKB-T23. Values are means ± standard error (s.e.) of FPKM. . '+' and '-' represent presence and absence of H3K4me3 marks, respectively.

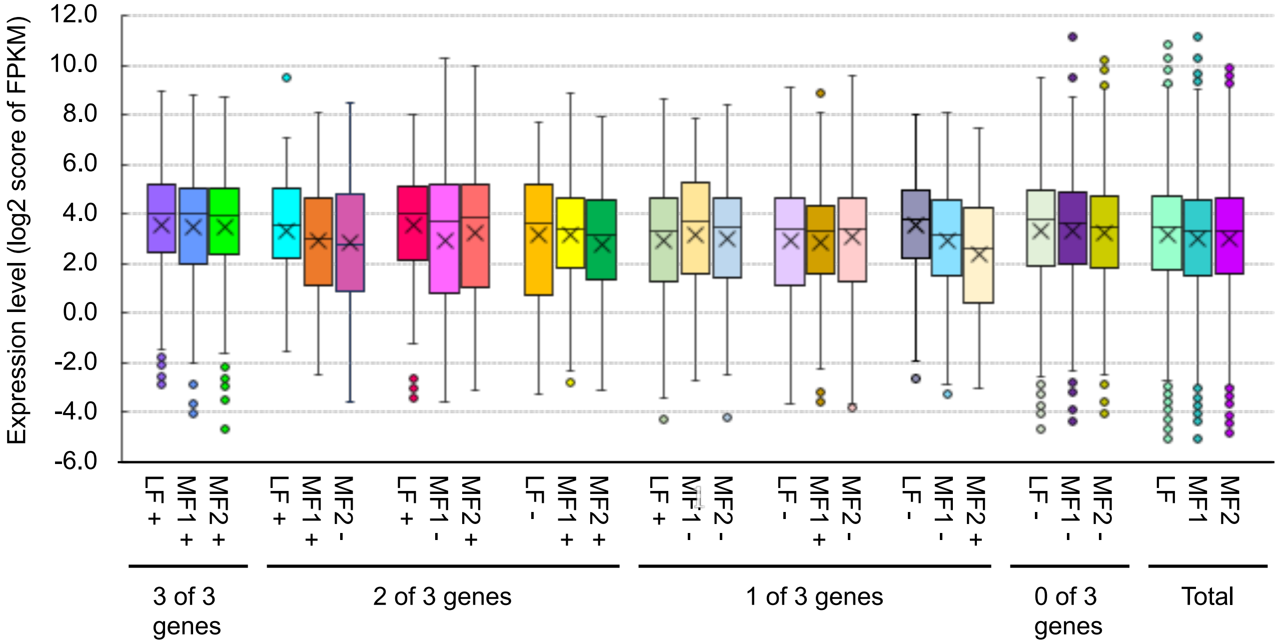

Supplementary Figure S12. Comparison of the expression level (FPKM) between paralogous pairs with and without H3K4me3 marks in RJKB-T24. Values are means ± standard error (s.e.) of FPKM. . '+' and '-' represent presence and absence of H3K4me3 marks, respectively.

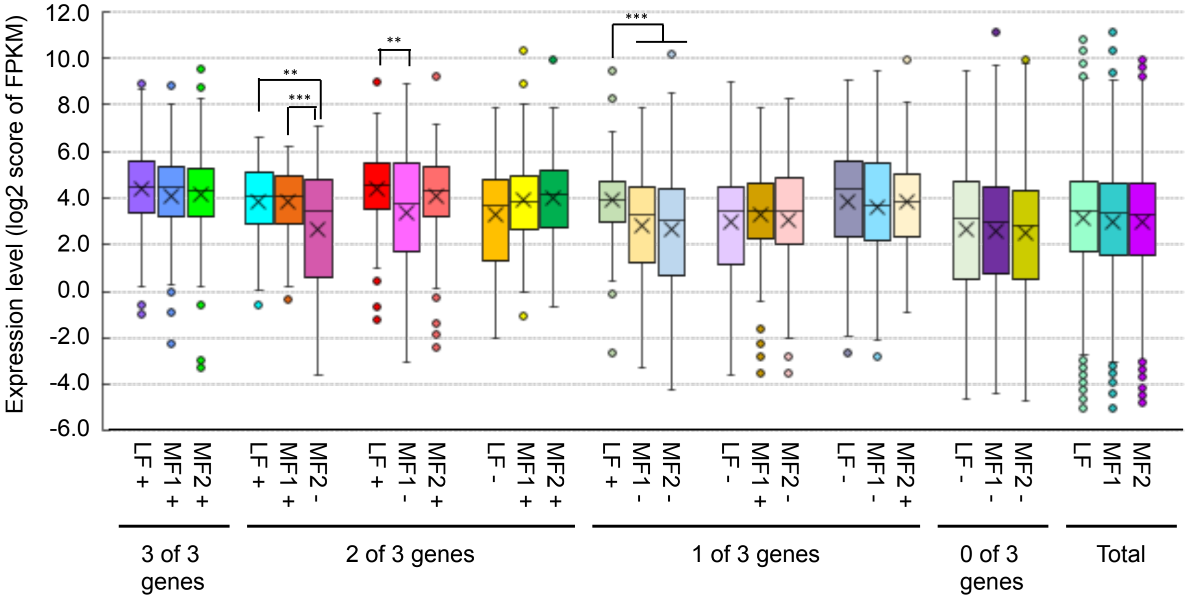

Supplementary Figure S 13. Comparison of the expression level (FPKM) between paralogous pairs with and without H3K36me3 marks in RJKB-T24. Values are means ± standard error (s.e.) of FPKM. . '+' and '-' represent presence and absence of H3K36me3 marks, respectively. **, $p < 0.01$; ***, $p < 0.001$ (Student *t*-test).

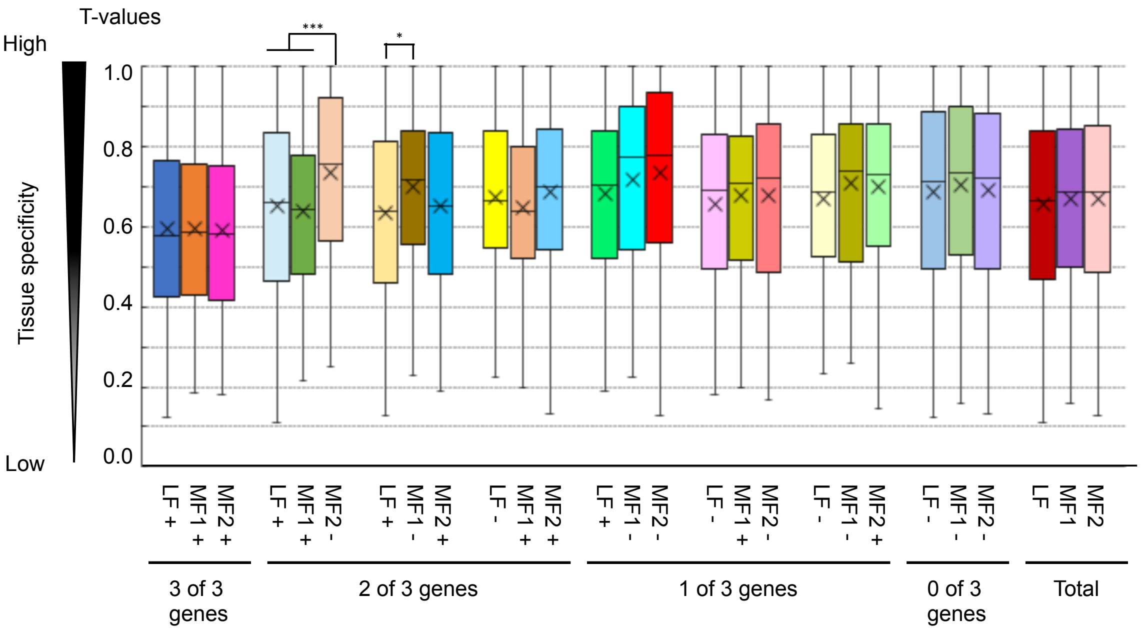

Supplementary Figure S 14. Comparison of the tissue specificity of expression (a tissue specificity index, T-value) between paralogous pairs with and without H3K4me3 marks. Values are means ± standard error (s.e.) of FPKM. '+' and '-' represent presence and absence of H3K4me3 marks, respectively. *, $p < 0.05$; ***, $p < 0.001$ (Student *t*-test).

Whole genome

	H3K4me3	H3K36me3	H3K9me2	H3K27me3	CG	CHG	CHH
H3K4me3		0.85	-0.23	0.28	-0.73	-0.60	-0.41
H3K36me3	0.87		-0.20	0.12	-0.62	-0.59	-0.45
H3K9me2	-0.25	-0.19		-0.15	0.48	0.35	0.21
H3K27me3	0.25	0.13	-0.17		-0.44	-0.28	-0.16
CG	-0.74	-0.64	0.47	-0.46		0.85	0.63
CHG	-0.61	-0.62	0.33	-0.30	0.85		0.86
CHH	-0.42	-0.48	0.19	-0.20	0.65	0.87	

Genic region

	H3K4me3	H3K36me3	H3K9me2	H3K27me3	CG	CHG	CHH
H3K4me3		0.64	0.30	-0.04	-0.21	-0.26	-0.18
H3K36me3	0.65		0.08	-0.11	0.00	-0.18	-0.15
H3K9me2	0.27	0.21		0.16	0.22	0.26	0.23
H3K27me3	-0.13	-0.18	0.14		-0.11	-0.05	0.00
CG	-0.19	0.04	0.24	-0.15		0.79	0.61
CHG	-0.26	-0.18	0.24	-0.06	0.75		0.79
CHH	-0.18	-0.15	0.21	-0.03	0.60	0.80	

IRRs

	H3K4me3	H3K36me3	H3K9me2	H3K27me3	CG	CHG	CHH
H3K4me3		0.79	0.51	0.56	-0.01	-0.02	-0.04
H3K36me3	0.78		0.53	0.45	0.03	0.00	-0.02
H3K9me2	0.40	0.56		0.57	0.15	0.12	0.04
H3K27me3	0.46	0.43	0.49		0.09	0.07	0.02
CG	-0.02	0.05	0.15	0.05		0.70	0.46
CHG	-0.03	0.02	0.11	0.04	0.71		0.62
CHH	-0.04	-0.01	0.04	0.01	0.47	0.63	

Supplementary Figure S15. The comparison between epigenetic states in RJKB-T23 (upper right) and RJKB-24 (lower left). The correlation coefficient of histone modifications quantified by reads per kilobase million (RPKM) and DNA methylation levels in each sliding window per 100 kb at the whole genome level. IRRs, interspersed repeats regions.

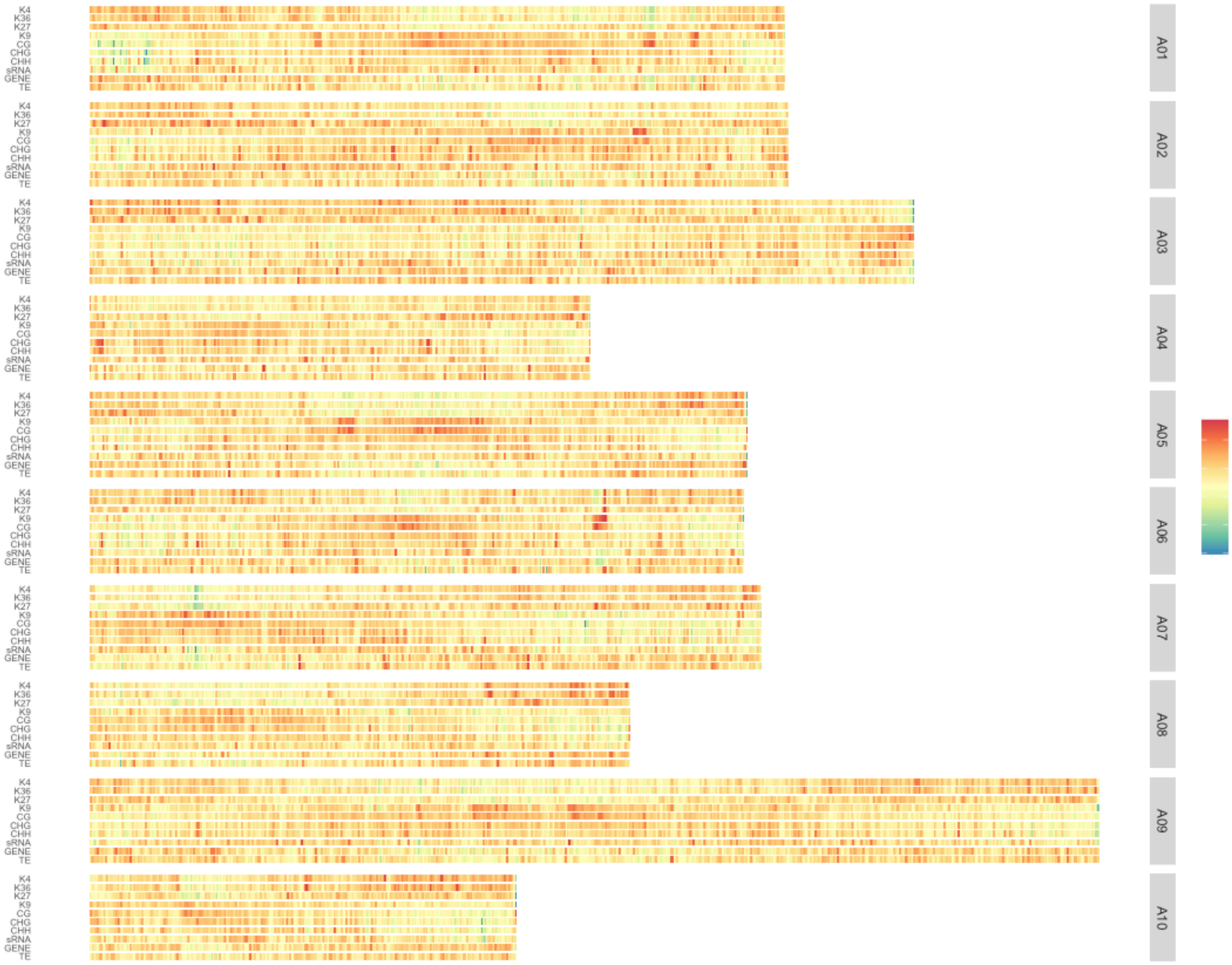

Supplementary Figure S16. The distribution of H3K4me3 and H3K36me3 and other epigenetic marks in RJKB-T23. Heatmap is visualized using ggplot2 package version 3.3.2 (https://ggplot2.tidyverse.org/) in R version 3.6.1 (https://www.r-project.org/). GENE and TE represent their expression levels.

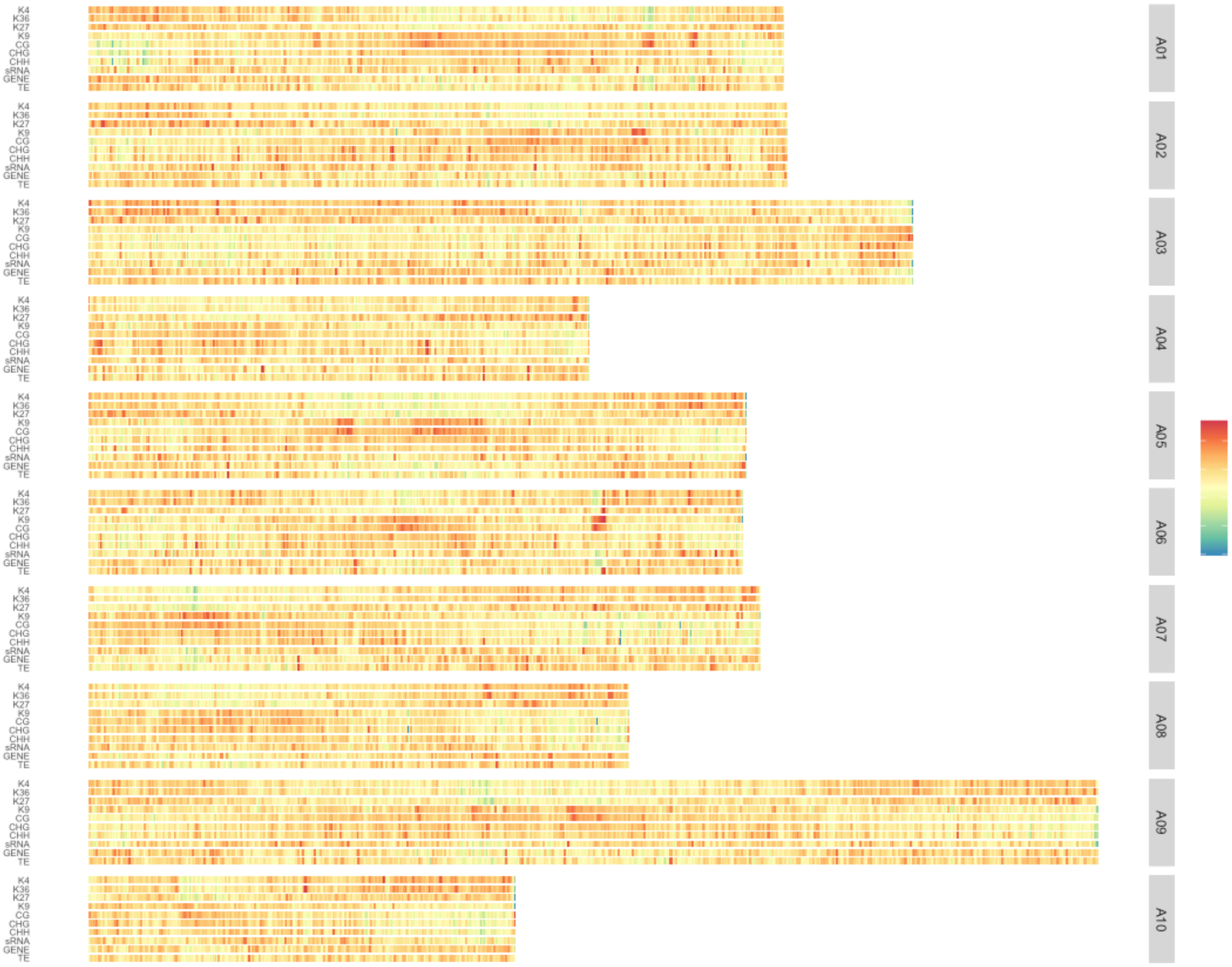

Supplementary Figure S17. The distribution of H3K4me3 and H3K36me3 and other epigenetic marks in RJKB-T24. Heatmap is visualized using ggplot2 package version 3.3.2 (https://ggplot2.tidyverse.org/) in R version 3.6.1 (https://www.r-project.org/). GENE and TE represent their expression levels.

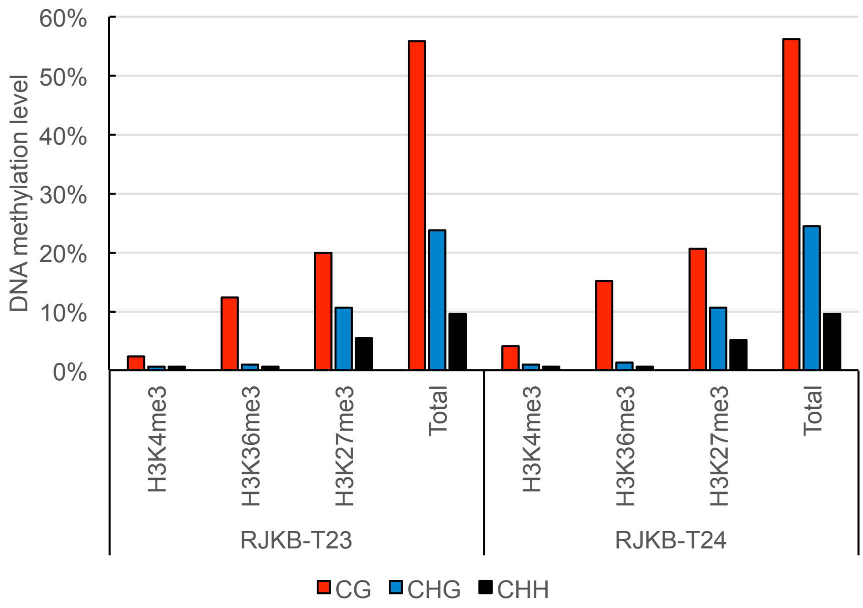

Supplementary Figure S18. DNA methylation level in the regions having H3K4me3 or H3K36me3 marks. ‘Total’ represents the average of DNA methylation levels in all regions of the genome.
